# Supplementary material for: Targeting Impaired Type I Interferon–IL-27 Signaling Rescues T Regulatory Cell Suppressive Function in Relapsing-Remitting Multiple Sclerosis
Source: bioRxiv. 2025 Aug 28:2025.08.26.671836. Preprint. [Version 1] doi: 10.1101/2025.08.26.671836 (PMC12954510; doi:10.1101/2025.08.26.671836)
Supplement: Supplement 1 [file media-1.pdf]

Table 1. (Study subjects for Figure 1.)

Subject	Age (years)	Sex	Race	Disease duration (month)	EDSS
MS1	39	F	C	2	2
MS2	30	M	AA	3	1
MS3	28	M	C	2	1
**average**	**32.3**	**1F, 2 M**	**1 AA, 2 C**	**2.3**	**1.3**

bject	Age	Sex	Race	Diagnosis
HC1	38	F	C	migraine
HC2	30	M	AA	vasovagal syncope
HC3	27	M	C	bipolar disorder
**average**	**31.7**	**1F, 2 M**	**1 AA, 2 C**	

Subject	Age (years)	Sex	Race	Disease duration (month)	EDSS
MS4	30	M	AA	3	1
MS5	28	M	C	3	1
MS6	37	M	C	2	0
MS7	34	F	AA	60	2
MS8	48	M	C	3	2
MS9	52	F	C	1	0
MS10	45	F	AA	120	0
MS11	33	M	C	1	1
MS12	24	F	AA	2	0
MS13	27	F	C	3	1
**average (n=10)**	**37.5**	**5 F, 5 M**	**4 AA, 6 C**	**24**	**0.8**

Subject	Age	Sex	Race	Control Diagnosis
HC4	30	M	AA	vasovagal syncope
HC5	27	M	C	bipolar disorder
HC6	38	M	C	functional neurological disorder
HC7	34	F	AA	migraine
HC8	48	M	C	chronic migraine, chronic pain syndrome
HC9	52	F	C	paresthesia, polyneuropathy
HC10	44	F	AA	chronic migraine, chronic pain syndrome
HC11	34	M	C	healthy donor
HC12	24	F	AA	headache
HC13	28	F	C	neck pain
**average (n=10)**	**36.7**	**5 F, 5 M**	**4AA, 6C**	

Table 2. (Study subjects for Figure 2.)

	Age (years)	Sex	Race	Disease duration (month)	EDSS	sample
MS14	43	M	AA	36	6.5	CSF
MS15	37	F	C	48	1.5	CSF
MS16	36	F	C	12	1	CSF
MS17	40	F	AA	24	2	CSF
MS18	52	F	C	6	2.5	CSF
MS19	41	M	AA	12	3.5	CSF
MS20	33	F	C	6	1.5	CSF
MS21	34	M	AA	12	1	CSF
MS22	43	F	AA	1	3	CSF
MS23	30	M	C	6	0	CSF
MS24	45	F	C	12	1	CSF
MS25	27	M	C	0.2	1	CSF
MS26	42	F	AA	12	1	CSF
MS27	38	F	C	2	2	CSF
MS28	50	M	C	12	2	CSF
**average**	**39.4**	**9 F, 6 M**	**6 AA, 9 C**	**13.4**	**2.0**	
MS29	48	M	AA	6	5	serum
MS30	34	M	AA	12	1	serum
MS31	41	F	NA	1	0	serum
MS32	43	M	C	18	2	serum
MS33	29	F	C	1	1	serum
MS34	30	F	C	2	1	serum
MS35	37	M	C	0.6	2	serum
MS36	33	F	C	2	3	serum
MS37	33	F	C	12	2	serum
MS38	37	F	C	0.6	1	serum
MS39	62	F	C	12	2	serum
MS40	47	M	C	6	2	serum
MS41	60	M	AA	48	1	serum
MS42	57	F	AA	120	2	serum
MS43	34	F	C	2	3.5	serum
MS44	49	F	C	5	2.5	serum
MS45	39	F	C	0.9	1	serum
MS46	38	F	C	1	0	serum

MS47	36	M	C	9	0	serum
MS48	38	F	C	2	2	serum
**average**	**41.3**	**13 F, 7 M**	**4 AA, 15 C, 1 NA**	**13.1**	**1.7**	

Subject	Age	Sex	Race	Control Diagnosis	sample
HC14	54	F	C	small vessel disease	CSF
HC15	56	F	AA	small vessel disease	CSF
HC16	40	F	C	migraine	CSF
HC17	47	F	C	migraine	CSF
HC18	71	F	C	altered mental status	CSF
HC19	28	F	C	intractable migraine	CSF
HC20	34	F	C	chronic pain syndrome	CSF
HC21	43	F	O	intractable migraines	CSF
HC22	41	F	C	Bell's palsy	CSF
HC23	65	M	C	end stage renal disease	CSF
HC24	56	F	AA	peripheral neuropathy	CSF
HC25	45	F	C	diffuse myalgias	CSF
HC26	42	F	C	chronic pain syndrome	CSF
HC27	33	F	C	chronic pain syndrome/ restless leg syndrome	CSF
HC28	55	F	C	peripheral neuropathy	CSF
HC29	38	F	C	hemisensory deficit, tingling	CSF
HC30	46	F	C	fatigue	CSF
HC31	53	F	C	stroke	CSF
HC32	62	F	AA	sensory polyneuropathy	CSF
HC33	29	F	C	migraine	CSF
**average (n=20)**	**46.9**	**19 F, 1 M**	**3 AA, 16 C, 1 O**		

HC34	46	F	C	sleep apnea, fatigue	serum
HC35	55	F	C	peripheral neuropathy	serum
HC36	34	F	C	right sided numbness, carpal tunnel syndrome	serum
HC37	38	F	C	facial and extremities tingling	serum
HC38	53	F	C	stroke	serum
HC39	30	F	C	urinary retention	serum
HC40	38	F	C	fibromyalgia rheumatica	serum
HC41	34	F	AA	headache	serum
HC42	43	F	C	migraine	serum
**average (n=9)**	**41.2**	**19 F, 1 M**	**3 AA, 16 C, 1 O**		

Table 3. (Study subjects for Figure 5.)

Subject	Age (years)	Sex	Race	Disease duration (month)	EDSS
MS49	49	F	C	6	3
MS50	42	M	C	2	2
MS51	43	F	AA	1	2.5
MS52	36	F	C	84	1
**average (n=4)**	**41.9**	3 F, 1 M	1 AA, 3 C	**23.3**	**2.1**

Subject	Age	Sex	Race	Control Diagnosis	
HC43	51	F	C	migraine	
HC44	40	M	C	subdural hematoma	
HC45	44	F	AA	migraine	
HC46	38	F	C	essential tremor	
**average (n=4)**	**40.2**	3 F, 1 M	1 AA, 3 C		

Table 1-3. Demographic data for MS patient and HC donors. EDSS, Expanded Disability Status Scale.

Table 4. (Reagents used in the study)

Human Antibodies				
**Antigen**	**Fluorochrome**	**Clone**	**Supplier**	**Identifier**
CD3	PE-Cyanine5.5	SK7	Invetrogen	35-0036-42
CD4	FITC	VIT4	Milteny Biotec	130-113-213
CD4	BV786	SK3	BD Biosciences	563877
CD25	APC	BC96	BioLegend	302604
CD127	PE/Cy7	A019D5	BioLegend	351320
CD25	APC	REA570 | 3G10	Milteny Biotec	130-133-749
CD127	PE	REA614 | A019D5	Milteny Biotec	130-113-414
**Mouse Antibodies**				
CD45	AF700	30-F11	eBioscience	6-0451-82
CD3	BV650	17A2	BioLegend	100229
CD4	FITC	RM4-5	eBioscience	11-0042-82
ICOS	Pe-Cy7	7E.17G9	ioLegend	117422
PD-1	PE	PC61	BioLegend	102008
Ly6C	APC	HK1.4	BioLegend	128016
IL-27	APC	MM27.7B1	BD Biosciences	562792
CD11b	PE	M1/70	BioLegend	101208
CD11c	BV421	N418	BioLegend	117329
CD80	PE	16-10A1	BioLegend	104708
CD86	BV786	GL-1	BioLegend	105043
IL-27RA	PE	W16125D	BioLegend	159004
TGF-β	BV421	TW7-16B4	BioLegend	141408
Granzyme B	PE	3G8.5	BioLegend	149704
TNF-α	BV605	MP6-XT22	BD Biosciences	569296
IFN-γ	BV605	XMG1.2	BioLegend	505840
IL-17A	Pe-Cy7	TC11-18H10.1	BioLegend	506922
LAG-3	BV421	C9B7W	BioLegend	125221
TIGIT	BV605	1G9	BioLegend	142121
GITR	BV421	DTA-1	BioLegend	126331
IL-10	PE	JES5-16E3	BD Biosciences	554467
CTLA-4	Pe-Cy7	UC10-4B9	BioLegend	106314
PD-1	BV605	29F.1A12	BioLegend	135220
Ki67	BV605	16A8	BioLegend	652413
FOXP3	APC	FJK-16s	eBioscience	17-5773-82

Helios	Pe-Cy7	22F6	BioLegend	137236
TIM-3	Pe-Cy7	RMT3-23	BioLegend	119716

Human Primers

Gene	Assay ID	supplier
GAPDH	Hs02786624_g1	ThermoFisher Scientific
MX1	Hs00895608_m1	ThermoFisher Scientific
ITGB7	Hs01565750_m1	ThermoFisher Scientific
ISG15	Hs01921425_s1	ThermoFisher Scientific
IFITM1	Hs00705137_s1	ThermoFisher Scientific
CD81	Hs01002167_m1	ThermoFisher Scientific
CD226	Hs00170832_m1	ThermoFisher Scientific
CD52	Hs00174349_m1	ThermoFisher Scientific

Mouse Primers

Gene	Assay ID	supplier
Actin B	Mm02619580_g1	ThermoFisher Scientific
ISG15	Mm01705338_s1	ThermoFisher Scientific
IFITM	Mm00850040_g1	ThermoFisher Scientific
OAS1A	Mm00836412_m1	ThermoFisher Scientific

Human ELISA kits

Kit	Supplier	Identifier
Human IFNb ELISA Kit	Antigenix America	RHF842CK
Human IL-27 ELISA Kit	R&D systems	NBP3-06784

Mouse ELISA kits

Kit	Supplier	Identifier
Mouse IL-10 Quantikine ELISA Kit	R&D systems	M1000B
Mouse IL-27 p28/IL-30 ELISA Kit - Quantikine	R&D systems	M2728

EAE Reagents

Kit	Supplier	Identifier
PLP (139-151)	ANASPEC	AS-63912
MOG (35-55)	Bio-Synthesis	CRB1000379
Pertussis Toxin from B. pertussis, Lyophilized in Buffer	List Labs	180
mject™ Freund's Incomplete Adjuvant (FIA)	ThermoFisher Scientific	77145
